# Editorial: The promise of immunogenetics for precision oncology

**DOI:** 10.3389/fonc.2023.1252189

**Published:** 2023-08-09

**Authors:** Elisavet Vlachonikola, Anton W. Langerak, Richard Rosenquist, Anastasia Chatzidimitriou

**Affiliations:** ^1^ Institute of Applied Biosciences, Centre for Research and Technology Hellas, Thessaloniki, Greece; ^2^ Department of Immunology, Laboratory Medical Immunology, Erasmus MC, Rotterdam, Netherlands; ^3^ Department of Molecular Medicine and Surgery, Karolinska Institutet, Stockholm, Sweden; ^4^ Clinical Genetics, Karolinska University Hospital, Solna, Sweden

**Keywords:** immunogenetics, precision oncology, immune receptors, tumor microenvironment, next generation (deep) sequencing (NGS)

The concept of precision medicine (PM) was introduced as early as the times of Hippocrates and later emphasized by Sir William Osler, both fostering the perception that “It is much more important to know what sort of patient has a disease than what sort of disease a patient has”. In the last 20 years, rapid advances in diagnostic technologies along with the development of novel targeted therapies have paved the way for the implementation of PM approaches in cancer treatment and prevention, based on the individual’s tumor characteristics, such as the genomic profile and the composition of the microenvironment of the malignant cells (1).

In the context of hematological malignancies, the distinctive features of the patients’ immune repertoires, namely the unique B-cell receptor (BcR) and/or T-cell receptor (TR) genetic rearrangements within their adaptive immune system, have evolved as highly promising tools for PM in patient stratification and disease monitoring. In the clinical setting, immunogenetics is the basis for clonality assessment which allows to chartacterize at the molecular level the architecture of a lymphoproliferation (2, 3). Additionally, immunogenetics has been proposed as a means of reliably assessing measurable residual disease (MRD) (4). Turning to translational research, immunogenetic analysis of immune repertoires has shed light on the complex biological heterogeneity of lymphoid malignancies, which is reflected in the observed clinical variability in everyday practice. Furthermore, accumulating evidence on intraclonal diversification of the immunoglobulin genes has further enhanced our understanding of the mechanisms driving disease ontogeny (5).

In this Research Topic of Frontiers in Oncology, a collection of ten papers provides different perspectives on the promise of immunogenetics in hemato-oncology, highlighting on the one hand the contribution of immunogenetics to the deeper and more precise understanding of the natural history of lymphoid malignancies, and on the other hand its value for tracking individual immunopathogenetic trajectories in hematological malignancies (**Figure 1**).

**Figure 1:**
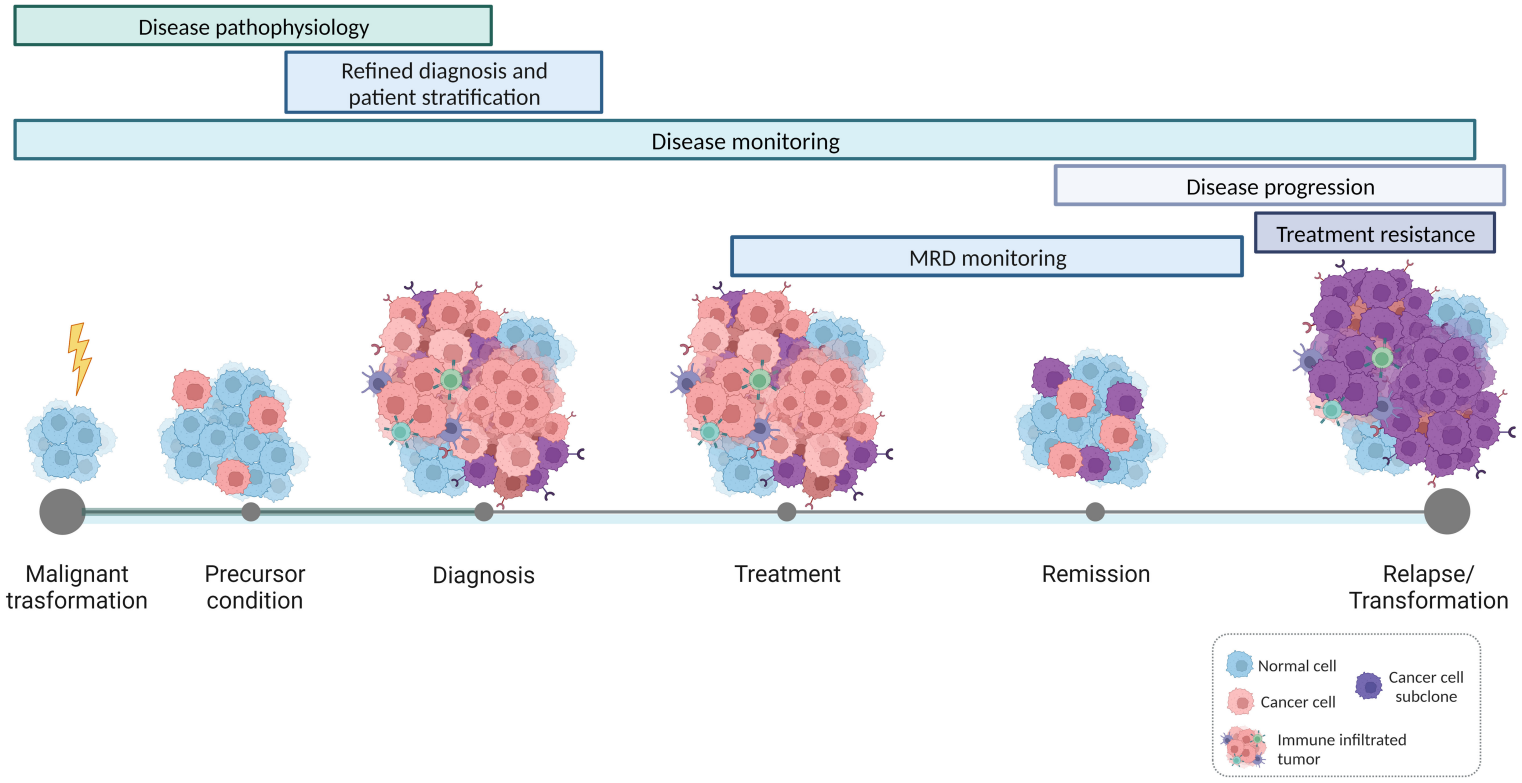
Applications of immunogenetics for precision hemato-oncology. High-throughput studies on the features and dynamics of antigen receptor gene repertoires contribute to a refined understanding of the natural history of lymphoproliferative disorders, shedding light on the mechanisms that drive malignant transformation and disease progression. In parallel, immunogenetics also offers valuable insights into the interactions occurring within the tumor microenvironment and the intricate communication between bystander immune cells and tumor cells. This, in turn, establishes the basis for patient-specific immunotherapeutic interventions.

In more detail, Michaeli et al. identify distinct mutational patterns of the clonotypic immunoglobulin heavy variable (IGHV) genes in longitudinal biopsies from patients with follicular lymphoma (FL) versus transformed aggressive FL (t-FL), compared also with healthy control germinal center (GC) samples. The authors highlight different signatures of clonal evolution and somatic hypermutation (SHM) in the FL tumor clones in sequential biopsies from the same patient, in which SHM leads to the accumulation of novel potential N-glycosylation sites. Along similar lines, two independent papers by Sofou et al. and Neuman et al. describe distinct selection processes that shape the malignant clone in patients with chronic lymphocytic leukemia (CLL) bearing unmutated or mutated IGHV genes, respectively. The findings discussed in both papers strongly attribute this feature to alterations in SHM mechanisms. Moreover, for the first time, intra-VH CDR3 variations due to SHM were documented within cases bearing ‘truly unmutated’ IGHV genes i.e. those lacking any SHM over the sequence of the rearranged, clonotypic IGHV gene. (Sofou et al.) As described by Gkoliou et al. distinct features in the IGHV gene repertoires and SHM profiles have also been observed in cases with multiple myeloma expressing IgA or IgG isotype, alluding to unique immune trajectories to disease development.

Considering the technical aspects of immunogenetics analysis, several publications within this Research Topic present different methodologies for capturing the immune repertoire diversity, while also offering novel insights into the value of early detection of clonal events in different hematological malignancies and their diagnostic implications. Groenen et al. provide a detailed description of the next-generation sequencing (NGS)-based clonality assay applied across a wide spectrum of entities, spanning from immunodeficiency and autoimmunity to lymphoma and solid tumors. The authors emphasize the contribution of NGS-based immunogenetics to the sensitive diagnosis and early distinction of malignant lymphoproliferations in complex clinical and histopathological contexts.

In a similar vein, Kolijn et al. document that immunogenetic profiling of pre-diagnostic samples could predict the transformation of primary Sjögren’s syndrome (pSS) lesions to B-cell lymphoma. They report that clonotypes of extranodal marginal zone lymphoma (eMZL) are detectable prior to overt eMZL diagnosis in the repertoire of activated B cells from pSS patients. Darzentas et al. present novel tools for the identification and characterization of clonal evolution of the malignant cells in B cell precursor acute lymphoblastic leukemia (BCP-ALL) with direct application for initial marker identification and MRD monitoring. As an alternative methodology for MRD detection in hematological malignancies, overcoming the demanding validation experiments and laboratory accreditation requirements of the NGS-based clinical tests, Schilhabel et al. present a robust and reproducible monitoring assay based on patient-specific, allele-specific oligonucleotide PCR, targeting the patient- and tumor-restricted clonally rearranged IGHV genes.

In-depth immunogenetic analysis of bystander immune cells within a tumor also provides valuable information regarding cell communication within the tumor microenvironment, thereby contributing to the development of patient-specific immune-based therapeutic interventions. In this Research Topic, Vlachonikola et al. describe restrictions in the TR gene repertoire in patients with CLL, likely arising from distinct genomic aberrations. This evidence supports the design of strategies to guide immune recognition by CLL-specific T cells as a complementary or alternative treatment in CLL.

While these manuscripts underline the huge potential of immunogenetics to transform patient management and cancer treatment, it is important to also acknowledge the limitations of the available techniques. Although NGS-based approaches offer great analytical depth, immunogenetic analyses typically rely on a single biopsy or a small sample, which may not fully capture the entire tumor’s complexity. To overcome this pitfall, high-resolution mapping at the single-cell level has been proposed as the upcoming breakthrough strategy, enabling the identification of novel biomarkers for disease monitoring and response to treatment. In this Research Topic, a review article focusing on the employment of single-cell technologies in CLL by Oder et al. describes in detail the current status of single-cell transcriptomics, genomics, epigenomics, immunogenetics and profiling of cellular subpopulations, of both malignant and non-malignant cells within the tumor microenvironment. Single-cell sequencing technology has already shown its potential in dissecting the complex landscape of CLL, with similar applications in other immune-mediated malignancies, however, as highlighted by the authors, several challenges still exist that must be addressed through an integrated multi-omics approach.

In summary, the articles in this Research Topic illustrate the vital role of immunogenetics in precision hemato-oncology by providing insights into the tumor’s intratumor heterogeneity and immunogenicity; the interaction between the tumor and immune system; and, potential targets for immunotherapies. By harnessing this knowledge, researchers and clinicians are entering a new era with more tailored and effective stratification and treatment schemes for patients with cancer.

## Author contributions

All authors listed have made a substantial, direct and intellectual contribution to the work, and approved it for publication.
